# Complement Receptor 2 Based Immunoassay Measuring Activation of the Complement System at C3-Level in Plasma Samples From Mice and Humans

**DOI:** 10.3389/fimmu.2020.00774

**Published:** 2020-05-05

**Authors:** Lene Halkjær, Anne Troldborg, Henrik Pedersen, Lisbeth Jensen, Annette Gudmann Hansen, Troels Krarup Hansen, Mette Bjerre, Jakob Appel Østergaard, Steffen Thiel

**Affiliations:** ^1^Department of Biomedicine, Faculty of Health, Aarhus University, Aarhus, Denmark; ^2^Department of Clinical Medicine, Faculty of Health, Aarhus University, Aarhus, Denmark; ^3^Department of Endocrinology and Internal Medicine, Aarhus University Hospital, Aarhus, Denmark; ^4^Department of Rheumatology, Aarhus University Hospital, Aarhus, Denmark; ^5^Department of Molecular Biology and Genetics, Aarhus University, Aarhus, Denmark; ^6^Steno Diabetes Center Aarhus, Aarhus University Hospital, Aarhus, Denmark

**Keywords:** complement activation, complement receptor 2, immunoassay, C3dg, iC3b, mouse, human

## Abstract

We aimed at establishing a sensitive and robust assay for estimation of systemic complement activation at complement component C3 level in mouse and human plasma samples. In order to capture the activation products iC3b and C3dg in a specific and physiological relevant manner we utilized a construct consisting of the iC3b/C3dg-binding site of human complement receptor 2 (CR2) attached to an Fc-part of mouse IgG. This construct binds C3dg and iC3b from both mice and humans. We purified the CR2-IgG construct from mouse B myeloma cell line supernatants, J558L-CR2-IgG, by protein G affinity chromatography. The CR2-IgG construct was used for capturing C3 fragments in microtiter wells and an anti-mouse or an anti-human-C3 antibody was used for detection of bound C3 fragments. Initially we tested the specificity of the assays with the use of purified C3 fragments. Further, with the use of the CR2-based assay, we measured an up to three-fold higher signal in activated mouse serum as compared to non-activated mouse serum, whereas activated serum from a C3 knock-out mouse gave no signal. We tested *in vivo* generated samples from a mouse experiment; complement activation was induced by injecting cobra venom factor or heat aggregated IgG into C57bl6 mice, followed by withdrawal of EDTA blood samples at different time points and measurement of iC3b/C3dg. We observed a clear time-dependent distinction in signals between samples with expected high and low complement activation. Furthermore, with the use of the assay for human C3 fragments, we observed that patients with systemic lupus erythematosus (SLE) (*n* = 144) had significantly higher iC3b/C3dg levels as compared to healthy individuals (*n* = 144) (*p* < 0.0001). We present two functional immunoassays, that are able to measure systemic levels of the C3-activation products iC3b and C3dg in mice and humans. To our knowledge, these are the first assays for complement activation that use a physiological relevant capture construct such as CR2. These assays will be a relevant tool when investigating mouse models and human diseases involving the complement system.

## Introduction

The complement system is an important part of the innate immune system and vital for maintenance of tissue homeostasis (1). Its primary task is to identify and opsonize targets including invading microbes, immune complexes, necrotic tissue and apoptotic cells and hereafter facilitate their removal (2). Traditionally complement activation occur through three different pathways; The classical, the lectin and the alternative pathway. Activation, through either of the three pathways, results in the cleavage of the central component of the system – the complement component C3 protein. From here onward the effector functions and end-products are the same: Opsonization by split products of C3, formation of the membrane attack complex, which lyses cells and the recruitment of inflammatory cells by pro-inflammatory mediators (1).

The enzyme cascades of the complement system are tightly regulated by a number of proteins (3, 4). If the delicate balance between activation and regulation is tipped; the system may act as a double-edged sword causing self-damage. The complement system has thus been suggested to play a crucial role in a variety of immune, inflammatory, neurodegenerative, ischemic and age-related diseases (5). To study the complement system and to analyze to which degree adverse activation of the complement system may play a role in diseases, a number of assays to measure systemic complement activation in human plasma samples exists. Some measure C3 and C4 with the assumption that low levels correspond to consumption due to activation (6). Others measure products generated by complement activation aiming at different properties of the activated forms than the native molecules; either by using monoclonal antibodies recognizing a neo-epitope in the molecule only present upon activation (7–10) or by using polyclonal antibodies after separation of larger native molecules from smaller activation products by polyethylene glycol (PEG)-precipitation (11). Another option is to study the total functional complement activity by, e.g., hemolytic assays (12).

In basic and translational research, mice are often the preferred *in vivo* model, but when it comes to studying mouse models of complement-related diseases and measuring systemic complement activation in mouse samples only a few assays exist (13–15). A reason for the relatively limited options could be the challenges scientists face, when aiming at establishing assays for murine complement activation, e.g., often monoclonal antibodies toward complement proteins are generated in mice and can therefore not be used in assays measuring mouse complement proteins (16, 17). Furthermore, separating smaller activation fragments from larger native proteins in mouse samples by, e.g., PEG is disadvantageous as Slp (sex-limited protein) found in mouse plasma seems to activate complement after PEG precipitation and thus possibly interfere with the estimated concentrations (18).

**FIGURE 1:**
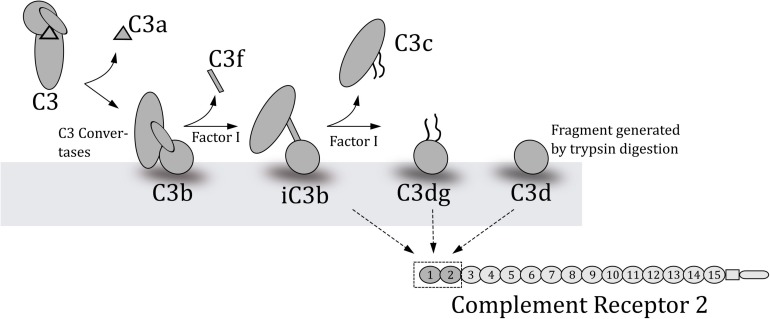
Schematic overview of the degradation of C3 to yield the various fragments of C3. C3b is created when C3 convertases cleave C3. Factor I in collaboration with co-factors cleaves the deposited C3b at several points to generate the indicated fragments. The lower part of the figure outlines which C3-fragments complement receptor 2 (CR2) binds and the two C-terminal domains where these fragments bind.

Consequently, in an attempt to establish an assay for complement activation in mouse samples, we aimed at another and more physiological relevant approach. We aimed at complement component C3 because it is the central molecule of the complement system, being an imperative part of all three initiating pathways. Upon activation of C3 through one of the pathways, a small fragment C3a is cleaved off C3. The remaining part, named C3b, undergoes an immense conformational change in the structure exposing new binding sites for other proteins (19) (Figure 1). During this change in structure a hidden thioester is exposed, which enables C3b to bind covalently to adjacent molecules on, e.g., tissue or microbial surfaces (20). Hereafter C3b can be further cleaved and processed by factor I aided by cofactors, generating iC3b and then C3dg (Figure 1). C3dg remains attached to the surface and may interact with complement receptors (21). The activation of the complement system is most efficiently initiated on surfaces, e.g., the initiators of the classical and the lectin pathways are active when found in clusters on a surface (22), but during such processes there is also a release of activation products into plasma and indeed the alternative pathway will also occur in solution (23). Such circulating products are what is measured in assays for activation products in plasma samples. Both C3dg and iC3b bind to complement receptor 2 (CR2), which is present on B lymphocytes and follicular dendritic cells (24) (Figure 1). Binding of the complement split products to CR2 lowers the amount of antigen necessary for activation of B cells (25). In humans, CR1 and CR2 are coded by two separate genes whereas the two complement receptors in mice are coded by the same gene (*Cr2*), but alternative splicing leads to two protein products – CR1 and CR2. Nonetheless, mouse CR2 is very similar in size and sequence to human CR2 (26).

Of the 15 short consensus repeats (SCRs) of the extra-cytoplasmatic region of CR2, only the two NH_2_-terminal repeats (SCR1-2) are required for iC3b and C3dg binding (27) (Figure 1). We therefore hypothesize that the C3dg/iC3b-binding parts of human CR2 can be used as a capture in an immunoassay measuring complement activation at C3-level in mouse and human samples. We decided to use a multivalent construct consisting of the C3dg/iC3b-binding site of human CR2(SCR1-2)_2_ attached to a Fc-part of mouse IgG. This construct binds C3dg and iC3b from both mice and humans and can be purified from culture media of a stably transfected mouse B myeloma cell line J558L (27). The purpose of this work was therefore to establish and validate a sensitive and robust assay for estimation of systemic complement activation at C3-level in mouse and human plasma samples based on a physiological relevant CR2-capture. Furthermore, we validated the assay with *in vivo* samples from both mice and humans.

## Materials and Methods

### Purification of CR2-IgG

The mouse B myeloma cells “J558L-CR2-IgG” (originally described in Hebell et al. (27) and kindly provided by professor M. Carroll, Boston) were cultured at 37°C at 8% CO_2_ in Dulbecco’s modified eagle medium (Lonza bioscience BE12 604F) supplemented with 10% fetal bovine serum with ultra-low IgG (Gibco 16250), 5 mM glutamax (Gibco 35050-038), 5 mM penicillin/streptomycin (Gibco 15140122), 50 μM mercaptoethanol (Sigma Aldrich M6250) and 0.6 mg/mL active G-418 (Sigma G8168). When the cells reached 1 × 10^6^ cells/mL they were spun down at 800 rpm for 7 min and frozen in storage medium [10% DMSO (Sigma D5879), 10% fetal bovine serum with ultra-low IgG (Gibco 16250) and 60% Dulbecco’s modified eagle medium (Lonza bioscience BE12 604F)]. The supernatants were withdrawn and added Na-Azid to 0.1%. Hereafter the supernatants were stored at 4°C until protein G purification.

To purify CR2-IgG from the supernatant we added Protein G beads (GE healthcare 17-06-18-01) buffered with PBS and mixed with 10 mM EDTA to avoid non-specific binding. The supernatants with protein G beads were left gently rotating at 20 rpm overnight at 4°C to allow for the binding of protein G to the Fc-part of mouse CR2-IgG. Hereafter the solution was spun at 700 rpm for 10 min leaving the CR2-IgG bound to protein G beads in the pellet. After withdrawing the supernatants, the beads were washed thoroughly with PBS, 10 mM EDTA and added to a column. After further wash, we added an elution buffer (0.1 M glycine-HCl, pH 2.5). Immediately after collection of fractions, the pH of the fractions was neutralized with 1 M Tris-HCL, pH 8.5. The protein concentration in the fractions was measured by spectrophotometry and the tubes containing protein were pooled (28).

### Immunoassay Measuring C3 Fragments in Mouse Plasma Samples

The complement activation products C3dg and iC3b, were measured in mouse EDTA-plasma samples by an in-house established sandwich-based TRIFMA-assay. Microtiter plates (Nunc MaxiSorb) were coated with the CR2-IgG construct at 5 μg/ml in PBS and incubated overnight at room temperature (RT). Residual binding sites were blocked by incubation for 1 h with 1 mg HSA/ml TBS followed by 3 times wash. All of the washing steps were made with TBS containing 0.05% Tween 20 (TBS/Tween). The standard curve was made by dilutions of mouse serum (Sigma-Aldrich, M5905). Mouse serum already contains activated C3 fragments. We chose this commercially available serum pool to allow others to obtain the same preparation if comparisons were to be done with other assays in other laboratories. The pool of mouse serum was defined to be 1 U/ml of C3dg+iC3b. The mouse serum was diluted 1/25 and further in seven sequential 2.5-fold dilutions in TBS/Tween, 10 mM EDTA with 1 mg HSA/ml and 0.1 mg heat aggregated human IgG per ml. HSA was added to the buffer to minimize background signal whereas the heat aggregated human IgG was added to avoid false positive signal from potential rheumatoid factors (RF) in the samples. Despite the known complement inhibiting effect of EDTA, the samples were kept on wet ice throughout the mixing and diluting procedures to avoid further in vitro complement activation. The samples were diluted 1/20 in the same buffer as described above. Three quality controls with a low, medium and high degree of complement activation were included for each plate to ensure tolerable inter-assay-variation (CV < 15%).

Test samples, quality controls and the standard were measured in duplicates, 100 μl per well and incubated overnight at 4 °C followed by wash. To detect bound C3dg+iC3b, we added 100 μl in-house biotinylated goat IgG anti-mouse complement C3 (MP Biomedicals cat. No. 55463) at 0.5 μg/ml in TBS/Tween with 1 mg HSA/ml and incubated at RT for 2 h. After wash with TBS/Tween we added 100 μl per well Eu^3+^-streptavidin (Delfia 1244-360, Perkin Elmer) diluted 1/1000 in TBS/Tween, 25 μM EDTA per well and incubated for 1 h at RT. After wash the signal was developed by adding 200 μl Enhancement buffer (Ampliqon laboratory reagents #Q99800). Plates were read with time-resolved fluorometry using a DELFIA-flurometer Victor5+ (Perkin Elmer) (Supplementary Figure S1).

In the process of establishing the assays several concentrations of the CR2-IgG-construct were tested as well as different biotinylated antibodies in different concentrations to find the optimal setup. The buffers were optimized during the process, especially in regard of lowering the background signal.

### Validation of the Assay for Murine C3 Fragments

In order to validate the assay, we tested three different types of mouse sera in wells coated with CR2-IgG as described above. We tested activated and non-activated mouse serum from C57bl6 mice and activated serum from a C3-knock-out mouse (29) (kindly provided by Dr. M. Carroll, Boston). Activation for both types of mouse sera was done as follows; 40 μl mouse serum was mixed with 1 mg/ml heat aggregated mouse IgG (#7404304, Lampire Biological Laboratories aggregated at 63°C for 1 h, 2 mg/ml) (activates the classical pathway) and 0.4 mg zymosan (Sigma #Z4250) (activates the alternative pathway) followed by incubation at 37°C for 4 h. Complement activation was stopped by addition of 2.2 μl 0.4 M EDTA and 1.6 μl Futhan (Sigma #N0289, 5 mg/ml H_2_O). All three types of sera were diluted 1/25 and in further three 3-fold dilutions in TBS/Tween 5 mM EDTA and added in duplicates as 100 μl per well. The signal was developed as described above.

We performed a gel permeation chromatography (GPC) fraction of serum to estimate the size of the molecules, which were detected in the assay. We analyzed both activated and non-activated mouse serum from C57bl6 mice. Sera (100 μl) were injected on a Superdex 200 increase 10/300 GL column (GE Healthcare). The running buffer was TBS/tween, 5 mM EDTA and fractions of 250 μl were collected in sterile deep (2.2 ml wells) 96-well plates (VWR cat. No. 732-2893). The fractions were diluted 1/2 in TBS/Tween 5 mM EDTA and added to plates coated with CR2-IgG at 5 μg/ml PBS and blocked by incubation with HSA. After incubation overnight at 4°C the signal was developed as described above.

We tested whether freezing and thawing of a sample would affect the concentration of C3-activation products measured in the assay by thawing and freezing aliquots of the same plasma sample 1 to 6 times. Furthermore, we tested the inter-assay variation by running three quality controls for every plate to make sure we had a tolerable inter-assay variation (CV < 15%).

### *In vivo* Validation of the Assay for Murine C3 Fragments

We measured plasma EDTA samples from mice expected to be high or low in C3-activation products. Eight male C57bl6 mice were injected with known activators of the complement system intravenously and plasma concentrations of C3-activation products were measured at different time points (the procedure was approved through license no. 2014-15-0201-00392). In order to minimize the number of mice, we chose to use only two for each stimulation. Plasma EDTA samples were obtained from all mice prior to the injections (*t* = 0 min). Hereafter we intravenously injected two mice with 1 μg cobra venom factor (CVF), two mice with 100 μg heat aggregated IgG, two mice with 500 μg heat aggregated IgG and two mice with PBS, the latter two mice acted as controls. We then withdrew plasma EDTA samples after 15, 60, and 120 min. At the time 120 min the mice were euthanized. During the experiment the mice were observed in order to ensure they were not suffering. To further ensure *ex vivo* inhibition of the complement system we added futhan to each EDTA plasma sample, to a final concentration of 50 μg/ml and kept samples on wet ice. For every mouse we measured the concentration of C3-activation products in the assay at the four different collection time points (*t* = 0, 15, 60, and 120 min.).

### Immunoassay Measuring C3 Fragments in Human Plasma Samples

The coating and blocking steps were done exactly as described for the mouse setup. The standard for the human assay was made by withdrawing serum from a healthy person, adding Na-Azid to 0.1%, and leaving it at 37°C for 1 week in the plastic tube, thereby gaining a pool of serum with a high degree of complement activation due to intrinsic activation (30). Dilutions of the standard were made analogous to the mouse setup and throughout the assay analogous buffers were used. Samples were kept on wet ice throughout the mixing and dilutions, to avoid in vitro complement activation. Three human serum samples with a low, medium, and high degree of complement activation were included as quality controls for each plate to ensure tolerable inter-assay-variation (CV < 15%).

The signal was developed in a TRIFMA-setup as described above for the assay for mouse C3 fragments, but instead of adding the biotinylated goat IgG anti-mouse complement C3, we added biotinylated rabbit anti-human C3dg (DAKO cat. No. A0063) at 0.5 μg/ml in TBS/Tween with 1 mg HSA/ml.

### Validation of the Assay for Human C3 Fragments

As for mouse serum, we tested dilutions of activated vs. non-activated human serum. The activated pool of serum was made by adding 1 mg/ml heat aggregated human IgG (#007815; CSL Behring GmbH, Germany, aggregated at 63°C for 1 h, 10 mg/ml) to human serum and then incubation at 37°C for 1 h. The activation was stopped by adding 10 mM EDTA. In addition, we performed GPC fractionation of activated and non-activated human serum analogous to the one performed and described for the mouse setup.

We tested three different purified forms of activated human C3, i.e., iC3b, C3dg, and C3c, in the assay to test the specificity of CR2-IgG toward C3dg and iC3b. iC3b was produced as previously described (31). C3dg and C3c was generated from C3b after cleavage with Factor I followed by separation of C3dg and C3c. To this end, C3b was mixed with Factor H (Complement Technologies) and Factor I (Complement Technologies) in a 1/100 and 1/500 mass ratio, respectively. The mix was incubated at 37°C for 1–2 day whilst C3dg generation was monitored by SDS PAGE. Upon incubation, the mix was diluted threefold in 20 mM HEPES (pH 7.5). To separate the C3dg from C3c, the mix was applied to a 1 mL Mono Q 5/50 GL (GE Healthcare) column and the protein was eluted by 60 ml linear gradient from 20 to 400 mM NaCl. The C3c was polished by size exclusion chromatography on a Superdex 200 increase (GE Healthcare) column equilibrated in 20 mM HEPES (pH 7.5), 150 mM NaCl. Similarly, the C3dg was purified on a Superdex 75 (GE Healthcare) column equilibrated in 20 mM HEPES (pH 7.5), 150 mM NaCl.

We coated wells with the CR2-IgG construct as described. We diluted the various C3 fragments in TBS/Tween, 1 mg HSA/ml and added them at three different concentrations; 5 μg/ml, 1 μg/ml and 0.2 μg/ml in duplicates, 100 μl pr. well. The signal was developed in a TRIFMA-setup as described above for the human setup.

We tested whether freezing and thawing of a sample affected the concentration of C3-activation products measured in the assay by freezing and thawing aliquots of two different human plasma samples 1–9 times. Serum, EDTA plasma, citrate plasma and heparin plasma were collected from three healthy persons to investigate the potential difference in C3dg+iC3b measurements using different collecting tubes. Furthermore, we tested the inter-assay variation by running three quality controls for every plate to make sure we had a tolerable inter-assay variation (CV < 15%).

### Validation of the Assay for Human C3 Fragments on Patients’ Samples

To validate the assay with *in vivo* samples, we measured the degree of complement activation within a cross-sectional cohort of patients with systemic lupus erythematosus (SLE) and healthy individuals. Blood samples from patients with SLE (*n* = 144) were collected at the Department of Rheumatology and blood samples from healthy controls (*n* = 144) at the Department of Clinical Immunology at Aarhus University Hospital (32). An overview of patients’ characteristics is found in the original article (32). Clinical investigations were conducted according to the Declaration of Helsinki. The Danish Data Protection Agency and The Regional Committee on Health Research Ethics approved the study (#1-10-72-214-13).

We matched the measurements of complement activation from our CR2-based immunoassay with measurements of complement activation from already established protocols. We had access to measurements of C3 and C3dg from the SLE patients (*n* = 143) (11), and measurements of C3d from some of the SLE patients (*n* = 58). The C3dg-immunoassay measures levels of C3dg in the supernatant obtained after a PEG-precipitation of other C3 related material (11), whereas the C3d assay is a double-decker rocket immunoelectrophoresis (33) used at hospitals in Denmark. The latter assay actually measures C3dg present in the plasma samples. The nomenclature C3d is a name previously used in such assays, as reactivity was confirmed by a C3d product produced by trypsin digestion of C3 fragments (see Figure 1).

### Statistics

The assumption of Gaussian distribution was tested with QQ-plots, histograms and F-tests. Data did not follow a normal distribution and log-transformation did not improve normality significantly. Data are therefore presented as median with interquartile range. To test for differences between groups a non-parametric Mann-Whitney test was performed. *P*-values < 0.05 were considered statistically significant. To compare measurements from our assay with measurements from established protocols we made spearman correlations and Bland-Altman plots. Stata version 15 and GraphPad Prism software package (version 7.0) were used for data management and statistical calculations.

## Results

### Validation of the Assay for Murine C3 Fragments

**FIGURE 2:**
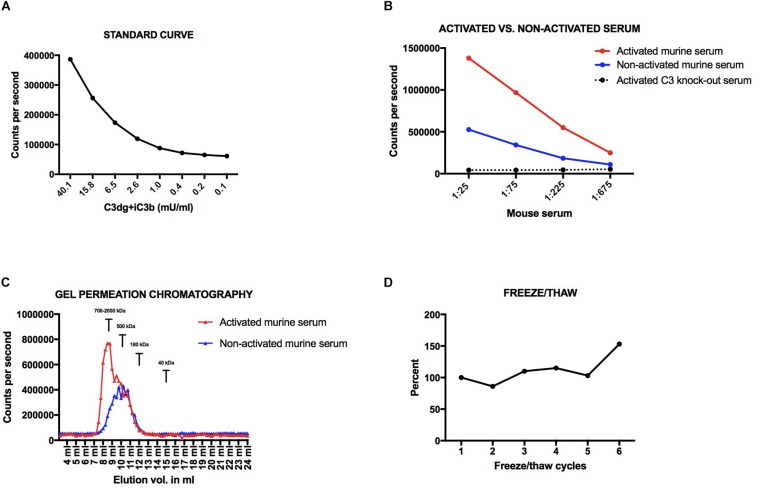
**(A)** Standard curve for the mouse assay. **(B)** Dilutions of activated and non-activated serum from a wildtype C57bl6 mouse and activated serum from a C3 knock-out mouse. **(C)** Activated vs. non-activated mouse serum on a superdex200 column. **(D)** Freeze-thaw cycles of the same mouse sample. Variations are presented in percentage with a starting point at 100%. All samples have been added to plates coated with CR2-IgG and the signal obtained after development using an anti-mouse C3 antibody.

We developed assays for estimation of the presence of activated forms of C3 in plasma – exploiting the iC3b/C3dg binding domains of CR2. The standard curve obtained in the assay for measurement of levels in murine samples is given in Figure 2A and we judged this to be acceptable in terms of signal to noise ratio and the dynamic range. In the following assay we included three quality controls to assure comparable measurements. The inter-assay variation was below 15%.

We found an up to three-fold higher signal in the activated serum as compared to the non-activated mouse serum (Figure 2B). The signal found in the non-activated mouse serum is most likely due to some spontaneous low-level activation of the alternative pathway generating C3-fragments. When measuring non-activated EDTA-samples, which are the preferred test samples, we see very low signals due to the complement-inhibiting effect of EDTA. Dilutions of activated serum from a C3 knock-out mouse gave no signals, which shows that C3 is necessary for signal in the assay, i.e., no other proteins are by themselves generating a signal, not even the aggregated IgG and zymosan added to the serum for activating the complement system (Figure 2B).

We analyzed the size of the molecules detected in the assay using size separation of serum samples on a GPC Superdex200 increase column. The majority of the signal in the activated serum sample eluted at 8–9 ml (Figure 2C). Based on standards of molecules with known molecular weights, an elution volume of 8–9 mL on this column corresponds to a size of approximately 700–2,000 kDa. This indicates that the assay detects C3-activation fragments attached to larger molecules, e.g., immune complexes. The majority of the signal of the non-activated serum sample, and a shoulder of the signal from the activated serum, eluted at 10–11 ml (approximately 500 kDa). No signal was seen at 12 ml where C3 (185 kDa) elutes on the column. In addition, we did not see a signal for free C3dg, which with a size of 36 kDa elutes at 15 ml. Nor did we see a signal for free iC3b, which should elute similar to the position of C3.

If we subjected mouse EDTA plasma to between 1 to 5 freeze/thaw cycles we did not see an increase in signal for C3dg+iC3b (CV < 15%), whereas we observed a 50% increase in concentration from cycle 5 to 6 (Figure 2D).

### *In vivo* Validation of the Assay for Murine C3 Fragments

**FIGURE 3:**
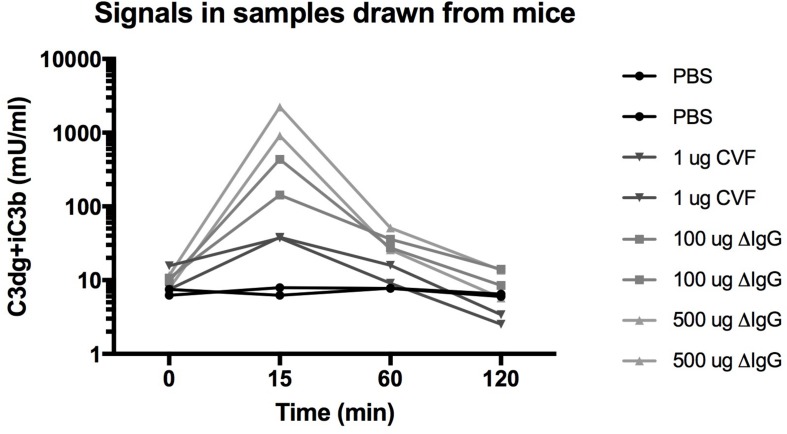
Measurements of C3-activation products in mU/ml in mouse EDTA plasma samples at time 0 before injection and at 15, 60, and 120 min after injections of either PBS, 1 μg cobra venom factor (CVF), 100 μg heat aggregated IgG, or 500 μg heat aggregated IgG.

We provoked complement activation in mice by injection of known activators of the complement system (aggregated IgG activating the classical pathway or CVF acting at the alternative pathway) and compared to injection of PBS (Figure 3). In EDTA plasma samples drawn from the mice we observe a concentration of C3dg+iC3b around 9.6 ± 3 mU/ml before intervention (*n* = 8). We observed no increase in C3dg+iC3b for the two mice that were injected with PBS (CV < 15% across the four time points). The six mice injected with the known stimulators of the complement system all showed increased concentrations of C3dg+iC3b after 15 min. For the two mice injected with CVF we observed an up to 5-fold increase in concentrations after 15 min. The largest increase in C3dg+iC3b fragments was observed after injections with heat aggregated IgG with an up to 45-fold (100 μg) vs. 180-fold (500 μg) increase after 15 min. At 60 min. the concentration of C3dg+iC3b had decreased for the mice that had received complement activating stimuli, and at 120 min the level was back to the signal seen at the starting point (Figure 3).

### Validation of the Assay for Human C3 Fragments

**FIGURE 4:**
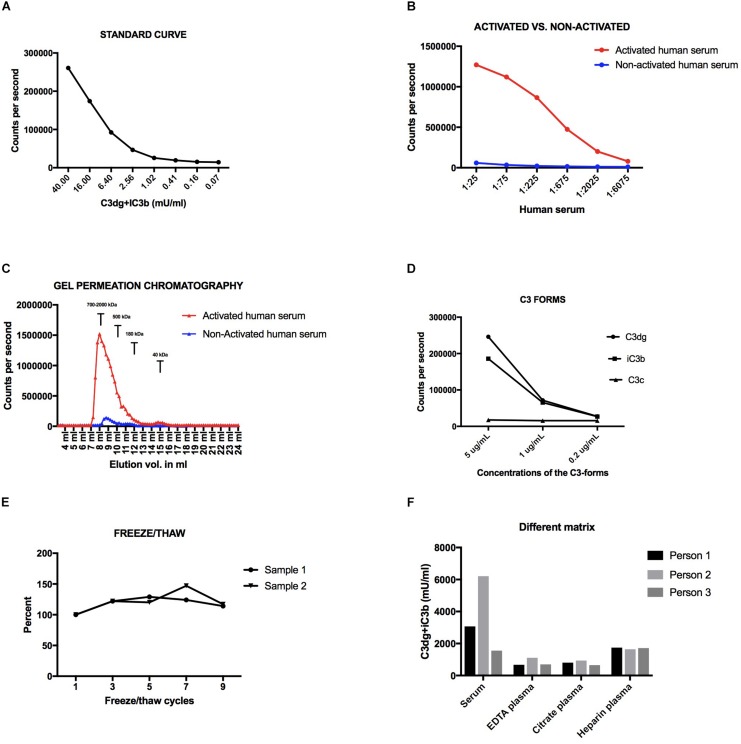
**(A)** Standard curve for the human assay. **(B)** Dilutions of activated and non-activated human serum. **(C)** Signals in fractions of activated and non-activated human serum after fractionation on a Superdex200 column. **(D)** Three different concentrations of three C3-forms (C3dg, iC3b, C3c). **(E)** Freeze-thaw cycles of two human samples. Variations are presented in percentage with a starting point at 100%. **(F)** Concentration of C3dg+iC3b in serum, EDTA-plasma, citrate-plasma and heparinized plasma in three persons. All samples have been added to plates coated with CR2-IgG and the signal obtained after development using an anti-human C3dg antibody.

The standard curve obtained in the assays for measurement of concentrations in human samples is given in Figure 4A and showed acceptable signal to noise ratio and dynamic range. As for the mouse setup, we included three quality controls to ensure comparable measurements. The inter-assay variation was below 15%. We measured an up to 20-fold higher signal in activated human serum as compared to non-activated human serum (Figure 4B). By size separation of serum samples, we observed a peak in signal corresponding to a size of approximately 700–2,000 kDa – similar to what we observed for mouse samples (Figure 4C). No peak was observed at 12 mL where human C3 or iC3b elutes from the column. For the activated human sample, we observed a peak at 15 mL corresponding to the size of free C3dg (36 kDa) (Figure 4C). Next, we tested three human C3-forms in the assay and observed higher signals for C3dg and iC3b as compared to no signal for C3c (Figure 4D). Freeze/thaw cycles, from 1 to 9 times, did not affect the concentrations measured by the assay in the human setup (CV < 15%) (Figure 4E). Concentrations of C3dg+iC3b varied depending on the collecting tubes. The highest signal was seen in serum samples as compared to EDTA plasma samples and citrate plasma samples, whereas heparinized plasma was intermediate or at the same level as the serum samples (Figure 4F).

### Assay for Human C3 Fragments on EDTA Samples From Patients With Systemic Lupus Erythematosus

**FIGURE 5:**
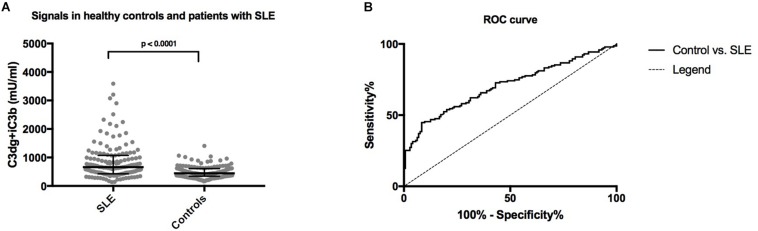
**(A)** A comparison of concentrations of plasma levels of C3dg+iC3b in systemic lupus erythematosus (SLE) patients and healthy controls measured by the human CR2-based assay. The non-parametric Mann–Whitney test was used for comparison. **(B)** ROC-curve for C3dg+iC3b comparing healthy controls and SLE patients.

Systemic lupus erythematosus patients showed significantly higher concentrations of C3dg+iC3b in EDTA plasma samples (median 662 mU/ml, IQR 428-1072) compared to healthy individuals (median 442, IQR 344-611) (*p* < 0.0001) (Figure 5A). Using a cut-off of 727.5 mU/ml, determined from the ROC-curve (Figure 5B) sensitivity, specificity and positive likelihood ratio (LR+) were determined to distinguish between the patient group and controls (Figure 5B). Comparing SLE to the healthy controls with the cut-off yielded a sensitivity of 44.8%, a specificity of 91.7% and a LR+ of 5.4.

We compared the results from our CR2-based assay with the results obtained in two other assays for C3 fragments as well as with the levels of C3 measured in the samples. We found a correlation to the two other assays that measure the degree of complement activation, but no correlation to the assay measuring full C3 (Supplementary Figures 2A,C,E). Using Bland-Altman plots (Supplementary Figures 2B,D,F) we show that our assay does not measure the exact same value as the other protocols and furthermore that the variation between the assays increased with increasing concentrations of complement activation products or C3.

## Discussion

Accumulating evidence suggest that dysregulation of the complement system may play an important role in a number of diseases (5, 34, 35). Logically this drives the development of strategies to specifically inhibit complement in anti-inflammatory therapy (36). To be able to progress in this field reliable methods to comprehensibly analyze the complement system are necessary. Especially assays that are able to measure the degree of complement activation are important in both animal and human studies of complement. Additionally, such assays, will aid the diagnosis of patients with possible complement-mediated diseases and allow the investigation and evaluation of potential new anti-inflammatory therapies targeting the complement system. We have established two immunoassays that may be used for determining complement activation in human or mouse samples. For both assays we utilize the specific binding sites of CR2 for capturing C3dg and/or iC3b. We believe that this reflects a physiological relevant step, as it resembles the *in vivo* situation where CR2 on B cells and follicular dendritic cells mediates recognition of complement reacted structures and thereby modulate the adaptive immune system.

As the biotinylated antibody tended to give high background signals in the assay for mouse C3 fragments, we added relatively high amounts of human serum albumin (HSA) to the buffer in order to reduce non-specific binding to the surface. HSA was also added in the dilution buffer for samples and standards to compete out non-specific binding of C3 to the surface, as serum/plasma contains high amounts of C3 (≈0.5–2 g/l). Such direct binding of C3 would lead to signal from the developing anti-C3 antibody. We added heat aggregated IgG to the buffers to avoid false positive signal from potential presence of RF in plasma. When analyzing clinical samples using sandwich-type immunoassays, a common – but not always recognized – problem is the presence of RF (37), i.e., autoantibody directed toward the Fc portion of IgG (38, 39). RF is known to be present in 80% of rheumatoid arthritis patients and 70% of Sjögren’s syndrome patients, but low levels of RF are present in virtually all individuals, i.e., the level of RF increases in general when the body is infected or vaccinated (40). The Fc portion of IgG is highly conserved in mammalians and RF in samples may influence assays and lead to false-positive signal in most sandwich assays, independently of the source of the antibodies employed. This happens if the RF directly cross-link the coated capture antibody and the developing antibody (41, 42). The heat aggregated IgG was added together with EDTA that chelates divalent metal ions and thus inhibits any in vitro activation of complement, i.e., activation of all the three pathways of the complement system are calcium and/or magnesium-dependent (3).

We evaluated the specificity of the coating construct by testing three human C3-fragments. The experiment outlines a clear binding of the CR2-IgG construct toward the C3-fragments; C3dg and iC3b. Moreover, we did not see any signals from C3 in fractions from serum samples separated by GPC. This is in line with several publications, which state CR2’s specificity toward C3dg and iC3b (24, 43, 44). Studies show that the binding site for CR2 in iC3b and C3dg lies within the common TED-domain (45, 46). Probably due to the homology between human and mouse CR2, the human C3dg/iC3b-binding domains of CR2 (SCR1-2) bind C3-activation fragments from both mouse and human. A technetium labeled recombinant version of the C3dg/iC3b-binding parts of CR2(SCR1-2) has previously been used to detect complement activation *in vivo* in mice in studies of ischemia/reperfusion injuries (47). We chose to use a multivalent fusion protein CR2(SCR1-2)_2_-IgG instead of a recombinant version of the C3dg-binding parts of CR2 for several reasons; First of all the dissociation constant for a monovalent interaction between CR2 and C3dg or iC3b is in the micromolar range indicating a need for a multivalent receptor (27). Second, when coating small molecules on a plastic surface a significant proportion of the molecules binding parts may be obscured. We hypothesized that coating with the bigger fusion protein CR2(SCR1-2)_2_-IgG would be advantageous in terms of sensitivity of the assays.

A major advantage of the assay presented here as compared to assays based on fractionating of larger native C3 forms from C3 fragments by, e.g., a PEG precipitation, is the possibility to measure C3 activation products bound to larger molecules. Both C3dg (36 kDa) and iC3b (174 kDa) carry the thioester site and are therefore most likely primarily found bound to other circulating plasma proteins and to a smaller extend found in their free forms (48, 49). Results from our GPC – for both mouse and human samples– confirm this, as the peak signals from the GPCs corresponds to a molecule size of approximately 700–2,000 kDa indicating that the majority of C3dg and iC3b in plasma is in complex with other molecules.

The ability of the assays to estimate C3dg+iC3b levels in EDTA plasma samples seemed stable as we observed acceptable inter-assay variation for both the murine and humane assay. Investigation of variations in the concentration of C3dg+iC3b in different matrixes indicated that samples collected in EDTA-coated tubes are preferred, which fortunately is the most common plasma sample type in biobanks. Furthermore, human EDTA plasma samples could be thawed and frozen up to 9 times without notable variations in C3dg+iC3b concentrations. A thorough study of the influence of freeze/thaw cycles on other parameters of complement activation is given in Yang et al. (50). We found that for mouse EDTA samples a similar signal was found for up to 5 cycles. For mouse samples, this emphasizes the need to handle samples in a similar way when comparing groups, even when EDTA is added and samples are kept on wet ice throughout the mixing and dilutions. A difference between mice and men in the composition and concentration of individual components of the complement system has been discussed by others (51) and may be a reason for the difference in stability through freeze/thaw cycles, i.e., that human samples may be more stable than mouse samples.

The validation with mouse samples generated from the *in vivo* activation experiment confirms that the assay is able to detect and distinguish samples with expected low and high complement activation. After intravenous injection of known activators of the complement system, the assay measures a significant increase in C3dg+iC3b in the plasma samples. The maximum concentration of C3dg and iC3b is seen 15 min after injection and declines hereafter. It can be speculated whether an even higher concentration of C3dg and iC3b would have been observed, if blood samples had been withdrawn earlier, or between 15 and 60 min after injection. We chose to withdraw only four blood samples from each mouse due to ethical considerations. It could be interesting to explore how fast complement activation happens in mice after injection to clarify, whether the activation is declining or rising at 15 min after injection. Furthermore, the rapid decline in C3dg+iC3b from 15 min to 60 min raises questions about the half-life of C3dg and iC3b. We could not find data on the half-life of iC3b and of C3dg in mice. In humans, iC3b has been reported to have a half-life of 90 min (52) and C3d (which we assume would be similar to C3dg) of 50 h (53). The latter half-lives of C3d in human plasma was estimated to be about 4 h in a patient undergoing an anaphylactoid shock (54). The half-lives of C3 fragments are summarized in Schramm et al. (10). Our experiment could indicate that the half-life of C3dg and iC3b in mouse plasma might be shorter than seen for the human body, but more experiments are needed to elucidate the half-life of C3dg and iC3b in mouse blood.

With regards to the measurements of complement activation in patient samples, we demonstrate that patients with SLE have significantly higher levels of C3 activation fragments as compared to healthy individuals. The value of the human assay we present in the present report as compared to other assays for complement activation specific fragments will have to be tested in several different patient cohorts. The different assays may indeed be useful in different settings (55, 56). Research has previously shown that measuring complement activation products in complement-related diseases like SLE could be advantageous in terms of sensitivity and specificity as compared to measuring low levels of C3 and C4, since the levels of these proteins are the net result of both consumption and synthesis (11, 57–59). However, when it comes to studying complement-related diseases in the preferred *in vivo* model – mice – low levels of C3 or C4 are still often used to estimate the systemic levels of complement activation (60). The established assay for mouse samples represent a novel opportunity to use a simple, stable and easy-to-use assay for complement activation products when performing studies on mice concerning complement related diseases. An advantage of the assay is that it is a great tool when doing translational research, as it can be used for both mouse and human plasma samples.

In the present report we use time-resolved immunofluorometric assays (TRIFMA) that utilizes europium as the label of the detecting reagent. In general, we find such assays to have a higher sensitivity and wider working range than the corresponding enzyme-linked immunosorbent assays (ELISA). We and others have experienced that the conditions used for the TRIFMA can easily be transferred to an ELISA format, i.e., exchange of the europium-labeled streptavidin for enzyme labeled streptavidin (11, 61).

In conclusion, we present two novel, stable and simple assays, which can measure complement activation in mouse and human samples. A range of assays for the estimation of activation of the human complement system have been developed (62). To our knowledge, the assays we present in this report, are the first to use a physiological relevant capture construct as CR2. Besides SLE, the assays will be a relevant tool when investigating a wide range of other diseases involving the complement system. The assays could be relevant when studying C3 glomerulopathy, paroxysmal nocturnal hemoglobinuria, atypical hemolytic uremic syndrome, age-related macular degeneration, as well as ischemia/reperfusion injury (55).

## Data Availability Statement

The datasets generated for this study are available on request to the corresponding author.

## Ethics Statement

The studies involving human participants were reviewed and approved by The Danish Data Protection Agency and The Regional Committee on Health Research Ethics (#1-10-72-214-13). The patients/participants provided their written informed consent to participate in this study. The animal study was reviewed and approved by The Danish Animal Experiments Inspectorate (license no. 2014-15-0201-00392).

## Author Contributions

LH, TH, JØ, and ST planned the studies. LH, AT, AH, and LJ performed the laboratory experiments. LH and JØ performed the animal experiment. ST, MB, and JØ supervised the laboratory experiments. AT was in charge of collecting clinical data and blood samples at AUH. HP developed the C3 fragments used to investigate the specificity of the construct. LH and ST wrote the initial manuscript and all authors participated in the editing of the manuscript.

## Conflict of Interest

The authors declare that the research was conducted in the absence of any commercial or financial relationships that could be construed as a potential conflict of interest.

## Supplementary Material

FIGURE S1 Immunoassay measuring complement activation products C3dg and iC3b. Wells of microtiter plates were coated with the CR2-IgG. Samples are added and the activated C3 fragments are bound. The bound fragments are detected with biotinylated anti-C3 antibody (anti-C3 in the case for the assay of mouse samples and anti-C3dg in the case of the assay for human samples) followed by Eu^3+^-streptavidin. The signal is developed by adding enhancement buffer and subsequently read by time-resolved fluorometry. The structure of the CR2 domains in complex with C3dg are adapted from Van den Elsen et al. (46).

FIGURE S2 Comparison of measurements using the presented (CR2-based) assay with other available assays used to assess complement activation. **(A)** Spearman correlation between measurements of C3dg+iC3b (CR2-based assay) and C3 (TRIFMA immunoassay) (*n* = 143). **(B)** Bland-Altman plot of C3dg+iC3b (CR2-based assay) and C3 (TRIFMA immunoassay) (*n* = 143). **(C)** Spearman correlation between measurements of C3dg+iC3b (CR2-based assay) and C3dg (PEG-based assay) (*n* = 143). **(D)** Bland-Altman plot of C3dg+iC3b (CR2-based assay) and C3dg (PEG-based assay) (*n* = 143). **(E)** Spearman correlation between measurements of C3dg+iC3b (CR2-based assay) and C3d (double-decker rocket immunoelectrophoresis protocol) (*n* = 58). **(F)** Bland-Altman plot of C3dg+iC3b (CR2-based assay) and C3d (double-decker rocket immunoelectrophoresis protocol) (*n* = 58). For the three correlation plots the spearman correlation coefficient r has been added together with the *p*-value testing the null hypothesis of no correlation. For the three Bland-Altman plots the 95% limits of agreement have been added as dotted lines. Note that the Bland-Altman plot for the comparison between the CR2 based assay and the C3d assay (based on double-decker rocket immunoelectrophoresis protocol) appears as a straight line of more or less 30 degrees. This is due to the fact that the unites for the C3d assay range from 22 arbs.enh/L for the lowest value to 103 arbs.enh/L for the highest value whereas the values for the CR2 based assay range from 136 mU/ml for the lowest value to 3591 mU/ml for the highest value. This results in an increasing difference with increasing values. We have added this Supplementary Figure S2F to make the three comparisons in the Supplementary Figure S2 equal.

## Abbreviations

CR1: Complement receptor 1
CR2: Complement receptor 2
CVF: Cobra venom factor
EDTA: Ethylene-diamine-tetra-acetic acid
Eu^3+^: Europium
GPC: Gel permeation chromatography
HSA: Human serum albumin
IQR: Interquartile range
PBS: Phosphate buffered saline
PEG: Polyethylene glycol
RF: Rheumatoid factor
Rpm: Revolutions per minute
RT: Room temperature
SCR: Short consensus repeats
SLE: Systemic lupus erythematosus
Slp: sex-limited protein
TBS: Tris buffered saline
TRIFMA: Time-resolved immunofluorometric assay.
