# Supplementary figures and images for: Complement Receptor 2 Based Immunoassay Measuring Activation of the Complement System at C3-Level in Plasma Samples From Mice and Humans
Source: Front Immunol. 2020 May 5;11:774. doi: 10.3389/fimmu.2020.00774 (PMC7214740; doi:10.3389/fimmu.2020.00774)

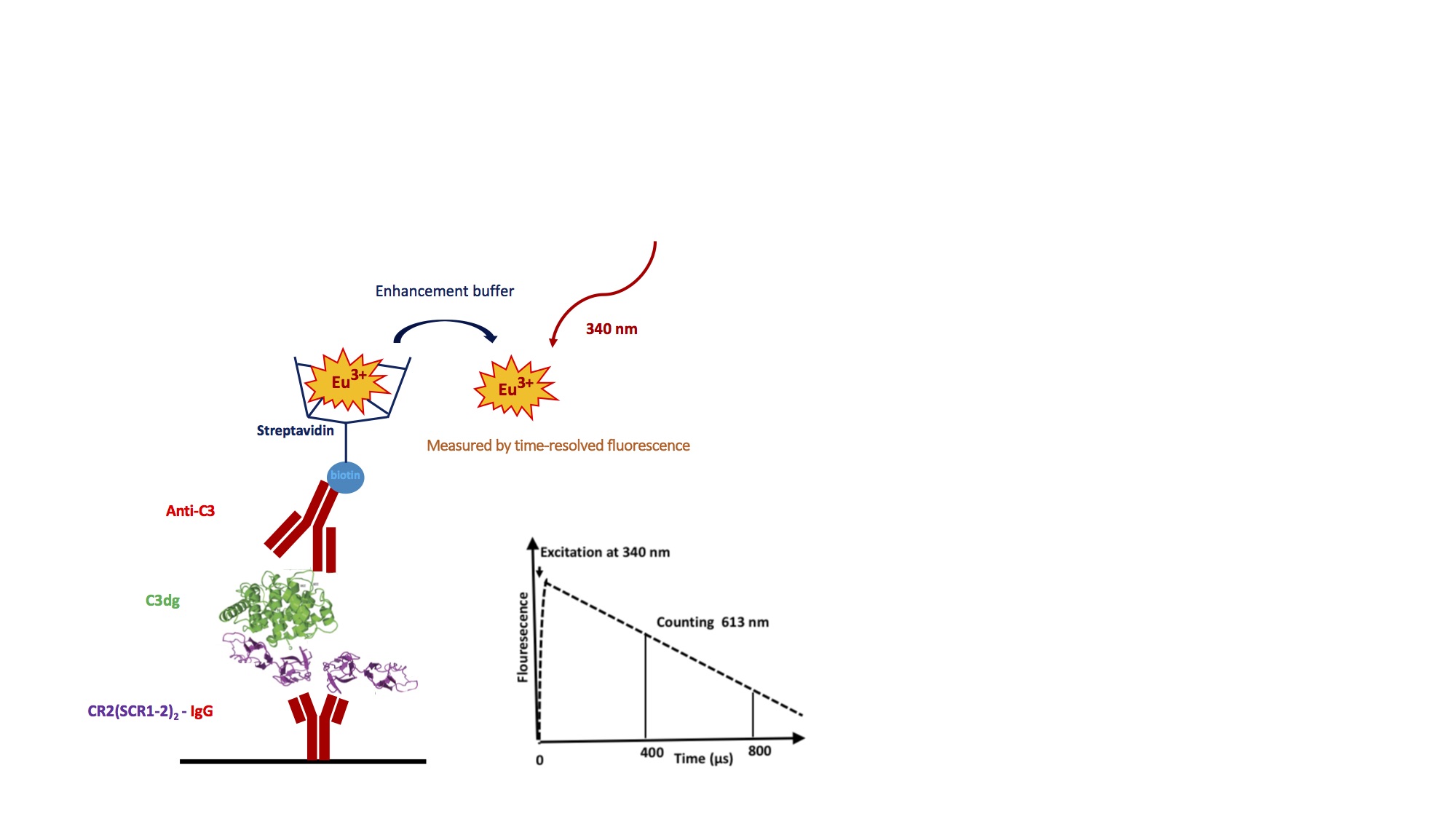

Supplement: FIGURE S1 — Immunoassay measuring complement activation products C3dg and iC3b. Wells of microtiter plates were coated with the CR2-IgG. Samples are added and the activated C3 fragments are bound. The bound fragments are detected with biotinylated anti-C3 antibody (anti-C3 in the case for the assay of mouse samples and anti-C3dg in the case of the assay for human samples) followed by Eu3+-streptavidin. The signal is developed by adding enhancement buffer and subsequently read by time-resolved fluorometry. The structure of the CR2 domains in complex with C3dg are adapted from Van den Elsen et al. (46). [file Image_1.JPEG]

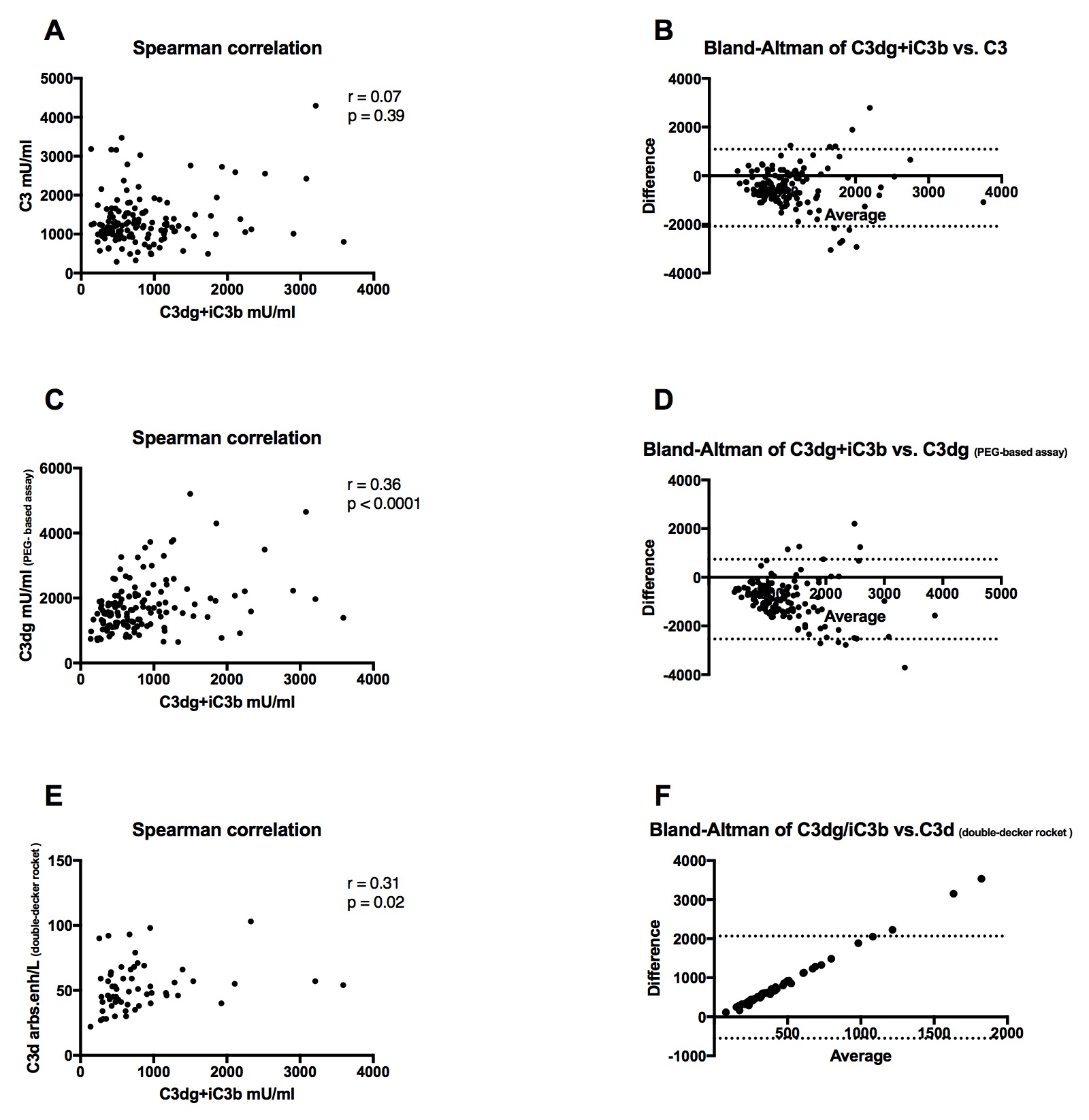

Supplement: FIGURE S2 — Comparison of measurements using the presented (CR2-based) assay with other available assays used to assess complement activation. (A) Spearman correlation between measurements of C3dg+iC3b (CR2-based assay) and C3 (TRIFMA immunoassay) (n = 143). (B) Bland-Altman plot of C3dg+iC3b (CR2-based assay) and C3 (TRIFMA immunoassay) (n = 143). (C) Spearman correlation between measurements of C3dg+iC3b (CR2-based assay) and C3dg (PEG-based assay) (n = 143). (D) Bland-Altman plot of C3dg+iC3b (CR2-based assay) and C3dg (PEG-based assay) (n = 143). (E) Spearman correlation between measurements of C3dg+iC3b (CR2-based assay) and C3d (double-decker rocket immunoelectrophoresis protocol) (n = 58). (F) Bland-Altman plot of C3dg+iC3b (CR2-based assay) and C3d (double-decker rocket immunoelectrophoresis protocol) (n = 58). For the three correlation plots the spearman correlation coefficient r has been added together with the p-value testing the null hypothesis of no correlation. For the three Bland-Altman plots the 95% limits of agreement have been added as dotted lines. Note that the Bland-Altman plot for the comparison between the CR2 based assay and the C3d assay (based on double-decker rocket immunoelectrophoresis protocol) appears as a straight line of more or less 30 degrees. This is due to the fact that the unites for the C3d assay range from 22 arbs.enh/L for the lowest value to 103 arbs.enh/L for the highest value whereas the values for the CR2 based assay range from 136 mU/ml for the lowest value to 3591 mU/ml for the highest value. This results in an increasing difference with increasing values. We have added this Supplementary Figure S2F to make the three comparisons in the Supplementary Figure S2 equal. [file Image_2.JPEG]
